# Impact of Varying Chest Wall Target Volume Delineation on Postmastectomy Radiation Therapy Outcomes in Breast Cancer Patients with Implant-Based Reconstruction

**DOI:** 10.3390/jcm12216882

**Published:** 2023-10-31

**Authors:** Pei-Yu Hou, Chen-Hsi Hsieh, Chen-Xiong Hsu, Deng-Yu Kuo, Yueh-Feng Lu, Pei-Wei Shueng

**Affiliations:** 1 Department of Radiation Oncology, Far Eastern Memorial Hospital, New Taipei 220216, Taiwan; jcgv03.be07@nycu.edu.tw (P.-Y.H.); chenci28@ym.edu.tw (C.-H.H.); cxhsu@mail.femh.org.tw (C.-X.H.); dykuo@mail.femh.org.tw (D.-Y.K.); d08456001@ntu.edu.tw (Y.-F.L.); 2 School of Nursing, Yuan Ze University, Taoyuan 320315, Taiwan; 3 School of Medicine, National Yang Ming Chiao Tung University, Taipei 112304, Taiwan; 4 Institute of Traditional Medicine, School of Medicine, National Yang Ming Chiao Tung University, Taipei 112304, Taiwan

**Keywords:** breast cancer, postmastectomy radiation therapy, implant-based reconstruction, target volume, dosimetry

## Abstract

Background: The target volume for post-mastectomy radiation therapy (PMRT) in breast cancer patients with reconstruction has been a subject of debate. Traditionally, the RT chest wall (CW) volume encompasses the entire implant. For patients with retropectoral implants, the deep lymphatic plexus dorsal part of the implant is no longer considered high risk and can be omitted. This study aimed to assess the radiation dose distribution and treatment outcomes associated with different CW delineation according to ESTRO ACROP guideline for patients who have undergone implant-based reconstruction. Methods: We conducted a retrospective review of breast cancer patients who underwent a mastectomy followed by two-stage implant-based breast reconstruction and adjuvant radiation therapy (RT) between 2007 and 2022. The expanders/implants were positioned retropectorally. The chest wall target volumes were categorized into two groups: the prepectoral group, which excluded the deep lymphatic plexus, and the whole expander group. Results: The study included 26 patients, with 15 in the prepectoral group and 11 in the whole expander group. No significant differences were observed in normal organ exposure between the two groups. There was a trend toward a lower ipsilateral lung mean dose in the prepectoral group (10.2 vs. 11.1 Gy, *p* = 0.06). Both groups exhibited limited instances of reconstruction failure and local recurrence. Conclusions: For patients undergoing two-stage expander/implant retropectoral breast reconstruction and PMRT, our data provided comparable outcomes and normal organ exposure for those omitting the deep lymphatic plexus.

## 1. Introduction

Breast cancer ranks among the most prevalent cancers affecting women worldwide, with an estimated 2.3 million new cases (11.7%) and ranking as the fifth most common cancer mortality (6.9%) in 2020 [1]. Although breast-conserving surgery is the primary approach for early-stage breast cancer, mastectomy and breast reconstruction have gained prominence as treatment options, particularly for advanced cases and cosmetic considerations. The timing of reconstruction can be immediate or delayed following mastectomy, and it can involve either autologous tissue or expander/implant-based techniques. Postmastectomy radiation therapy (PMRT) has significantly reduced locoregional recurrence, overall recurrence, and breast cancer-related mortality, particularly in advanced and lymph node (LN)-involved breast cancer cases. However, radiation therapy (RT) can lead to complications in breast reconstruction patients, such as capsular contracture, implant loss, and fat necrosis, due to radiation-induced damage to skin and soft tissues, affecting blood flow and fibroblasts [2].

The choice between immediate breast reconstruction (IBR) and delayed breast reconstruction (DBR) hinges on various factors, including the surgeon’s expertise and the need for further oncologic treatments like adjuvant radiation therapy (RT). DBR is often favored when PMRT is necessary to mitigate associated reconstruction complications. However, a growing trend in the United States is to recommend IBR even for patients requiring PMRT. As reported for reconstructive practices in the United States [3], a quarter of surgeons seldom recommend delayed reconstruction and about 65% recommending immediate expander/implant reconstruction. Several systemic review and prospective cohort studies have shown satisfactory outcomes and similar complication rates with DBR [4,5,6,7].

Despite the diversity in surgical procedures, there is a discrepancy in defining the RT target volume for patients with implants. In contemporary cancer care emphasizing personalized RT, a more consistent and specific RT consensus can enhance oncologic treatment outcomes while minimizing side effects. The 2019 ESTRO ACROP consensus addressed this issue for postmastectomy implant-based reconstruction RT, offering distinct RT target volume recommendations based on the implant’s position [8]. During the breast reconstruction procedure, the implant (tissue expander or permanent implant) may be positioned either prepectorally (above the pectoralis major muscle) or retropectorally (below the pectoralis major muscle). Following a mastectomy, most local recurrences tend to occur in the skin and subcutaneous tissue, where residual glandular tissues and draining lymphatics are predominantly found. As a result, the target volume for PMRT on the chest wall should encompass the lymphatic plexus region situated between the skin and the pectoralis major muscle. Traditionally, the RT chest wall volume encompasses the entire implant. However, for patients with retropectoral implants, only the subcutaneous lymphatic plexus ventral to the implant/pectoral muscle is considered the high-risk region and should be irradiated. The implant and the deep lymphatic plexus situated dorsally to the pectoral muscle is no longer considered high risk and can be omitted from the target volume. Consequently, the RT volume comprises only the tissue rim ventral to the pectoralis muscle and the implant, forming a curved shape and reducing the irradiated implant volume. This approach differs significantly from the chest wall volume used for patients with a prepectoral implant, which includes both the ventral and the dorsal part of the implant in the RT volume, essentially irradiating almost the entire implant. The hypothesis that reducing the irradiated chest wall/implant volume could result in less exposure of normal organs or have an influence on reconstruction failure rates requires verification.

In our institution, we adopt a two-stage expander/implant breast reconstruction procedure with retropectoral implant insertion for patients requiring subsequent RT. This study retrospectively reviews the clinical outcomes, reconstruction complications, and RT planning of breast cancer patients who underwent postmastectomy reconstruction and adjuvant RT. We aim to explore the implications of distinct RT target volume delineation according to the ESTRO ACROP consensus.

## 2. Materials and Methods

### 2.1. Patient Population

We conducted a retrospective review of breast cancer patients who underwent mastectomy with reconstruction at Far Eastern Memorial Hospital (FEMH) in Taiwan between 2007 and 2022. Immediate implant-based reconstruction following mastectomy was performed for patients not requiring adjuvant RT. For patients indicated for adjuvant RT, a two-stage expander/implant-based breast reconstruction approach was adopted. This involved initial mastectomy surgery with temporary expander implantation, followed by partial inflation with saline during the RT course. Permanent implant reconstruction was subsequently performed at least six months after completing RT. Retropectoral implant placement was used for patients needing PMRT. Indications for adjuvant RT included pathological LN involvement, clinical LN involvement before neoadjuvant therapy, and positive margins. This study received approval from the Human Experimentation Committee of FEMH (FEMH-111179E).

### 2.2. RT Treatment Plan

Radiation volumes comprised the clinical tumor volume (CTV) encompassing the chest wall (CW) with or without regional nodal irradiation (RNI) of supraclavicular and infraclavicular regions, any portion of the at-risk axillary bed, and optional internal mammary nodes (IMN). Patients were divided into two groups based on their RT chest wall target volume. The prepectoral group included the CW covering the area between the skin and the pectoral muscle, omitting the expander and deep lymphatic plexus. The whole expander group included the entire expander and the deep lymphatic plexus in the CW. The planning target volume (PTV) was defined as the CTV plus a 5–8 mm margin for setup error. RT prescription doses were conventional schedules of 45–50.4 Gy in 25–28 fractions or hypofractionated doses of 40–42.5 Gy in 15–16 fractions with daily fractions. An additional boost dose was permitted for the surgical scar or gross lesions. RT techniques included hybrid RT, intensity-modulated RT (IMRT), and modern arc techniques with either volumetric modulated arc therapy (VMAT) or helical tomotherapy (HT).

### 2.3. Reconstruction Complication

We recorded reconstruction complications, including capsular contracture, implant failure, fat/muscle necrosis, infection, and wound dehiscence. 

### 2.4. Statistical Analysis

Patient characteristics, treatment factors, and dosimetry parameters were analyzed and compared between the two groups using the Mann–Whitney U test for continuous variables given the smaller sample size and the Chi-square test for categorical variables. If the limited patient numbers did not meet the Chi-square test criteria, then Fisher’s exact test was used for categorical variable comparison. Results were considered statistically significant when the *p*-value was less than 0.05. Statistical analysis was performed using SPSS software version 28.0 (SPSS Inc., Chicago, IL, USA).

## 3. Results

### 3.1. Demographics

**Figure 1:**
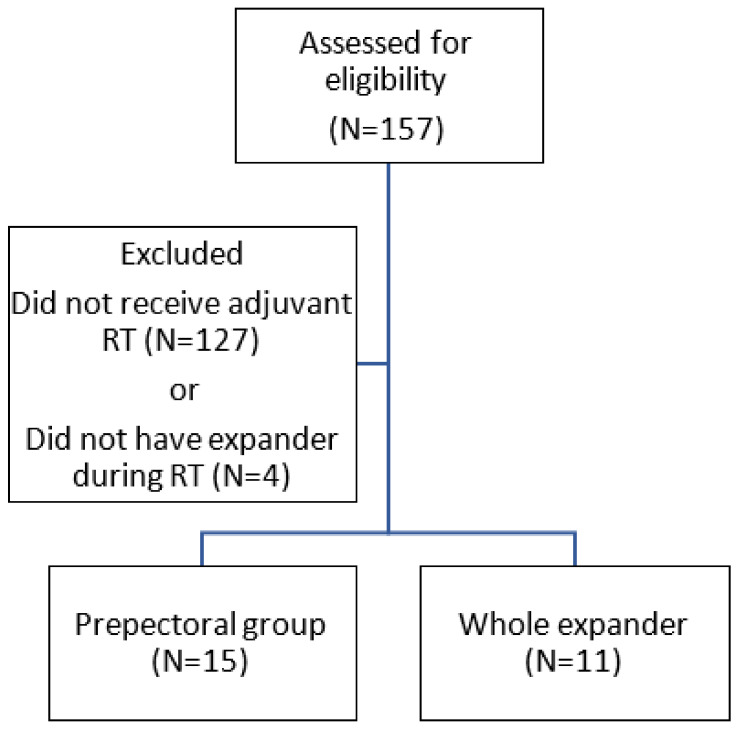
Patient enrollment. Abbreviations: RT—radiation therapy.

**Figure 2:**
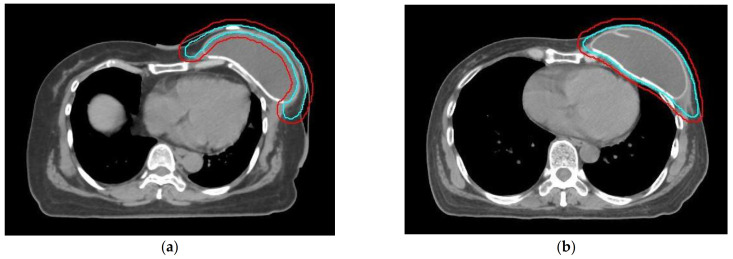
This figure illustrates the distinct chest wall (CW) target volume for the two groups, denoted as CTV (bright blue) and PTV (red). (**a**) Prepectoral group: The target volume encompasses the area between the skin and the pectoral muscle. Only the subcutaneous lymphatic plexus located ventrally to the implant is irradiated. (**b**) Whole expander group: The entire expander and deep lymphatic plexus is irradiated as the CW target volume.

Between January 2007 and December 2022, 157 breast cancer patients underwent radical, modified radical, or total (simple) mastectomy with expander/implant-based breast reconstruction. After excluding those who did not receive adjuvant RT and those who had had their prostheses removed before RT delivery, 26 eligible patients with breast cancer were enrolled. All expanders were retropectorally positioned. Among these patients, 15 had RT chest wall target volumes covering the area between the skin and the pectoral muscle (prepectoral group), excluding the expander. The other 11 patients had their chest wall target volumes include the entire expander (whole expander group). The patient enrollment process is shown in Figure 1. No significant differences were observed in terms of age, smoking history, histology, tumor grade, cancer stage, biomarkers, or systemic therapy usage between the two groups. Detailed patient characteristics are presented in Table 1. The difference in chest wall delineation is shown in Figure 2.

### 3.2. Comparisons of Dosimetric Outcomes

The median RT dose administered was 50 Gy, with a range of 40–61 Gy. No statistically significant differences were found in terms of RT technique, total dose, number of fractions, or the use of conventional or hypofractionation schedules between the prepectoral and whole expander groups. The RT treatment factors and dosimetry parameters are listed in Table 2. In the comparison of dosimetry parameters between the two groups, similar exposure doses were observed for the normal organs, including the heart, ipsilateral lung, and contralateral breast. However, there was a notable trend towards a lower mean dose to the ipsilateral lung in patients in the prepectoral group (10.2 vs. 11.1 Gy, *p* = 0.06).

### 3.3. Clinical Outcomes

The median follow-up period was 3.7 years. Reconstruction failure was observed in only one patient, a 48-year-old woman with right breast cancer. She had no history of smoking. Her initial cancer status was cT3N2M0, with ER-positive, PR-positive, and HER-2-positive tumors. She received neoadjuvant systemic treatment, including chemotherapy and anti-HER-2 targeted therapy. In November 2017, she underwent a modified radical mastectomy (MRM) with tissue expander-based reconstruction. The final pathology report indicated ypT3N1M0 disease. She had completed the adjuvant chemotherapy, anti-HER-2 target therapy, RT with prepectoral CW target volume, and anti-hormone therapy. In October 2018, approximately 11 months after the initial reconstruction, a permanent implant was inserted. However, approximately two months later, she developed complications, including pectoral muscle necrosis and wound dehiscence. Upon re-operation, avascular pectoral muscle was observed beneath the non-healing wound, leading to the removal of the implant. 

Additionally, one patient in the whole expander group experienced local recurrence. This 30-year-old woman had left breast cancer initially diagnosed as cT4aN1M0, and her tumor was triple-negative. She underwent neoadjuvant systemic therapy followed by MRM with breast reconstruction. The final pathology revealed ypT2N1M0 disease. She completed adjuvant chemotherapy and received PMRT with a dose of 60.4 Gy. However, approximately four months after completing RT, she experienced disease progression with local recurrence on the chest wall and distant metastases. 

## 4. Discussion

In our institution, breast cancer patients planning to undergo PMRT receive a two-stage expander/implant-based reconstruction with retropectoral implant insertion. The delineation of the PMRT chest wall target volume has been revised in some cases following the publication of the ESTRO ACROP contouring consensus guideline in 2019. This consensus provides recommendations for the context of PMRT after immediate implant-based reconstruction for breast cancer [8]. The delineation varies based on the position of the expander/implant, whether retropectoral or prepectoral. According to the consensus, the CTV_chest wall should only include the ventral part of the implant in cases where only the subcutaneous lymphatic plexus requires irradiation. Omitting the deep lymphatic plexus has the potential to improve the therapeutic ratio, but the impact of this de-escalation RT policy on outcomes needs further evaluation. 

As our preliminary study reports, there are two groups with different target volumes, prepectoral and whole expander, and no significant differences were observed in terms of exposure to normal organs, including the ipsilateral lung, contralateral breast, and heart. Only a trend toward a lower mean dose to the ipsilateral lung was noted in the prepectoral group (10.2 vs. 11.1 Gy, *p* = 0.06), although it did not reach statistical significance. Reconstruction failure and local recurrence rates were limited in both groups. This could be related to the relatively small breast reconstruction size in Asian women, resulting in no significant changes in exposure to adjacent normal organs with a reduction in the target volume. To the best of our knowledge, there is no other study analyzing the dosimetry of different chest wall contouring according to the ESTRO consensus for PMRT after implant-based reconstruction. Further research involving different racial groups and countries worldwide may provide more evidence on this matter.

There has been a significant increase in the trend of post-mastectomy breast reconstruction over the past decade in high socioeconomic societies, including the United States, Europe, North and South Asia, and Australia [9,10,11,12]. In Korea, the rate of post-mastectomy breast reconstruction increased after coverage by the National Health Insurance Service, with the majority opting for implant-based reconstruction. According to a multidisciplinary expert panel consensus, the two-stage technique is appropriate if implant-based reconstruction is planned, especially in the setting of PMRT [2]. The choice of reconstruction procedures should be tailored to individual breast cancer patients, considering both oncologic and aesthetic outcomes. However, there are limited data and varying opinions on determining the optimal RT methods for prepectoral and retropectoral reconstruction with regard to clinical outcomes and complication rates. Although the capsular contracture rate is higher in the prepectoral group (52.2 vs. 16.1%, *p* = 0.0018) with more severe contractures [13], some studies report similar reconstruction complication rates between the two populations [14]. Following the ESTRO ACROP recommendation to exclude the deep lymphatic plexus dorsal to the implant or pectoralis muscle in some cases, further evaluation of reconstruction complications and oncologic outcomes is needed over a longer follow-up period.

In the context of immediate reconstruction with a two-stage expander/implant procedure, the target volume for PMRT may encompass either the temporary expander or the permanent implant. In our patient group, expanders were partially inflated during the RT course. The issue of the expander/implant volume for PMRT has been debated. According to a multidisciplinary expert consensus, any manipulation involving inflation or deflation of the tissue expander before RT is discouraged [2]. The global 2021 oncoplastic breast consortium expert panel also provided inconclusive opinions regarding whether full expansion of the expander is necessary [15]. Additionally, modern RT techniques, such as IMRT, VMAT, and helical tomotherapy, can improve PMRT target coverage and dose distribution. The presence of an expander or a permanent implant does not compromise RT delivery. Retrospective data evaluating PMRT with VMAT techniques for tissue expander-based reconstructions showed a low reconstruction failure rate (4.3%). The inflation volume of the expander before PMRT did not affect the planning target volume or normal organ exposure (heart, lung, contralateral breast) except for the skin, which experienced an increased dose with larger expander volumes [16].

A population-based cohort study in Canada enrolled patients who had undergone mastectomy with immediate reconstruction [17]. Patients who received PMRT had increased risk of reoperation for breast reconstruction, regardless of the type of reconstruction: either implant-based or flap-based. Certain characteristics and dosimetry parameters increased the risk of reconstruction complications in the PMRT setting, such as smoking, PTV V107 > 11%, and the use of bolus [18]. Another study focused on breast cancer patients undergoing mastectomy, planned two-stage reconstruction and PMRT, and analyzed the association between bolus use for skin dose coverage and reconstruction complications. They reported that, for 288 consecutive patients, daily bolus use increased reconstruction complications such as infection and expander loss, especially in the second stage of reconstruction (4–5% vs. 10–12% for infection and implant loss, respectively) [19]. Generally, patients underwent the first stage of surgery before RT, and the second stage of surgery was performed at least several months after postmastectomy RT. The influence of RT on irradiated soft tissue would affect perfusion and wound healing, potentially contributing to more complications in the second stage. A study examining the dose–response relationship between reconstruction complication risk and dosimetry parameters demonstrated the role of D2cc or 0.03 cc in the reconstructed breast or overlying breast skin and boost in the setting of two-stage expander/implant reconstruction [20]. Further research to analyze the association between dose–volume histograms (DVH) and reconstruction complications to validate these findings is ongoing. 

The optimal sequence of mastectomy, reconstruction, and RT in two-stage prosthetic reconstruction or PMRT for a tissue expander or permanent implant remains a subject of debate. High-quality studies addressing this issue are eagerly awaited. For instance, a study by Naoum et al. explored PMRT timing and modality associated with complications in two-stage expander/implant reconstruction [21]. They found that early PMRT to a temporary expander before exchange to a permanent implant increased reconstruction failure, requiring prosthesis removal, compared to PMRT to a permanent implant. However, 5-year local control rates were similar (95–97%). Proton therapy was associated with higher capsular contracture and overall reconstruction failure compared to photon therapy. Another study involving 257 consecutive patients investigated the effects of PMRT on temporary expanders or permanent implants, revealing a higher total reconstruction failure rate in the population receiving PMRT to temporary tissue expanders (40% vs. 6.4%, *p* < 0.0001) [22]. The results regarding capsular contracture rates, shape and symmetry assessments by surgeons, and patient opinions favored PMRT to permanent implants.

Long-term outcomes of prosthetic-based reconstruction at Memorial Sloan Kettering Cancer Center yielded different results. The study showed greater predicted 6-year reconstruction failure rates for patients receiving RT to tissue expanders compared to those receiving RT to permanent implants (32% vs. 16.4%, *p* < 0.01). However, patients receiving RT to permanent implants experienced more moderate to severe capsular contracture (*p* < 0.01) and worse aesthetic results (*p* < 0.01) [23]. A meta-analysis of implant-based reconstruction and the timing of PMRT for more than 2000 patients from 2000 to 2016 arrived at a similar conclusion. It identified a higher reconstructive failure rate when PMRT was applied to tissue expanders compared to PMRT applied to permanent implants (20% vs. 13.4%, *p* = 0.008) but a lower capsular contracture rate (24.5% vs. 49.4%, *p* = 0.08) [24]. In a prospective multicenter longitudinal cohort study investigating PMRT to temporary expanders or permanent implants, no differences in any complication, major complication, or reconstructive failure were observed between the two groups. The overall reconstructive failure rate was 10.7%, and the timing of PMRT was not a predictor of complications [25].

A survey revealed that when patients underwent immediate breast reconstruction and planned to receive PMRT, more than half of plastic surgeons favored implant-based approaches [26]. However, outcomes of expander/implant or autologous reconstruction in the PMRT setting have shown that the expander/implant-based procedure had more complications requiring reoperation and reconstruction failure than autologous reconstruction in a median 8-year long-term follow-up [5]. Certain characteristics, such as a body mass index ≥ 30, smoking, diabetes mellitus, and hypertension, were significant predictors of reconstruction complications.

There are some limitations to this study. It is retrospective and was conducted at a single institution, relying on the institutional clinical practice experience of breast surgeons, plastic surgeons, and radiation oncologists, with a relatively short follow-up period. The limited number of cases is related to heterogeneous patient characteristics and dosimetry parameters in RT planning, which may have impacted the results. Since the impact of de-escalating RT regimens on patient outcomes requires further evaluation, the statistical power of this study (obtained from 26 patients) is limited. Most patients in this study received modern arc techniques such as VMAT or helical tomotherapy. Further exploration of distinct RT target volumes and associated clinical and dosimetry outcomes will provide more information to determine the optimal therapeutic approach for patients undergoing breast reconstruction and PMRT. The impact of different expander inflation volumes during the RT course on RT dosimetry planning, especially for patients whose chest wall target volume omits the deep lymphatic plexus, is an issue and may influence outcomes.

## 5. Conclusions

Currently, there is a lack of high-quality evidence to establish a consensus on the optimal approach to breast reconstruction, sequence, timing, RT target volume, and RT technique for patients planning to receive PMRT. For patients undergoing two-stage expander/implant breast reconstruction with retropectoral insertion, our data have provided preliminary outcomes and dosimetry comparisons of PMRT between prepectoral and whole expander irradiated volumes. We observed similar exposure to normal organs, particularly a trend toward a lower ipsilateral lung dose, and comparable local control for those omitting the deep lymphatic plexus.

## Figures and Tables

**Table 1:** Patient characteristics.

Characteristics	Prepectoral (n = 15)	Whole Expander (n = 11)	*p*-Value
Age (SD) (range)	50 (7.6) (32–58)	50 (11.6) (27–65)	0.61
Rt side	9	6	0.78
Lt side	6	5	
Pathology			
IDC	15	10	0.42
ILC	0	1	
Grade			0.95
Gr1 + 2	7 (1 + 6)	5 (1 + 4)	
Gr3	8	6	
Stage			0.11
Stage 1 + 2	5 (0 + 5)	8 (1 + 7)	
Stage 3 + 4	10 (9 + 1)	3 (3 + 0)	
pTis + T1 + T2	13 (0 + 2 + 11)	10 (1 + 2 + 7)	1
pT3 + T4	2 (2 + 0)	1 (1 + 0)	
pN0 + 1	6 (1 + 5)	8 (2 + 6)	0.13
pN2 + 3	9 (6 + 3)	3 (3 + 0)	
Clinical or pathological M1	1	0	1
ER positive	12	9	1
PR positive	12	7	0.41
HER-2 positive	4	2	1
Chemotherapy	15	10	0.42
Target therapy	4	2	1
Anti-hormone therapy	12	8	1
Immunotherapy	1	0	1
Smoking	2	1	1

**Table 2:** RT treatment factors and dosimetry parameters.

Characteristics	Prepectoral (n = 15)	Whole Expander (n = 11)	*p*-Value
RT technique			0.61
Hybrid + IMRT	3 (2 + 1)	1 (0 + 1)	
VMAT + gelical tomotherapy	12 (8 + 4)	10 (5 + 5)	
RT total dose (Gy) (SD) (range)	50 (5.4) (45–60)	55 (6.9) (40–61)	0.26
Number of fractions (SD) (range)	25 (3.2) (20–33)	30 (5.2) (15–33)	0.22
Conventional fractionation: N (%)	14	10	1
Hypofractionation: N (%)	1	1	
Heart mean dose (Gy) (SD) (range)	2.8 (1.67) (0.74–6.36)	2.64 (4.35) (1.02–14.98)	0.72
Ipsilateral lung mean dose (Gy) (SD) (range)	10.2 (2.86) (3.54–12.72)	11.1 (3.75) (6.76–19.72)	0.06
Contralateral breast mean dose (Gy) (SD) (range)	2.89 (1.19) (0.82–5.05)	2.47 (1.44) (1.32–6.13)	0.92

Abbreviations: RT—radiation therapy; IMRT—intensity-modulated radiation therapy; VMAT—volumetric modulated arc therapy; HT—helical tomotherapy; SD—standard deviation.

## Data Availability

The data presented in this study are available on request from the corresponding author. The data are not publicly available due to patients’ privacy and medical ethics.

## References

[1] Sung H., Ferlay J., Siegel R.L., Laversanne M., Soerjomataram I., Jemal A., Bray F. (2021). Global cancer statistics 2020: GLOBOCAN estimates of incidence and mortality worldwide for 36 cancers in 185 countries. CA A Cancer J. Clin..

[2] Nava M.B., Benson J.R., Audretsch W., Blondeel P., Catanuto G., Clemens M.W., Cordeiro P.G., De Vita R., Hammond D.C., Jassem J. (2019). International multidisciplinary expert panel consensus on breast reconstruction and radiotherapy. J. Br. Surg..

[3] Momoh A.O., Griffith K.A., Hawley S.T., Morrow M., Ward K.C., Hamilton A.S., Shumway D., Katz S.J., Jagsi R. (2020). Post-mastectomy breast reconstruction: Exploring plastic surgeon practice patterns and perspectives. Plast. Reconstr. Surg..

[4] Billig J., Jagsi R., Qi J., Hamill J.B., Kim H.M., Pusic A.L., Buchel E., Wilkins E.G., Momoh A.O. (2017). Should immediate autologous breast reconstruction be considered in women who require post-mastectomy radiation therapy? A prospective analysis of outcomes. Plast. Reconstr. Surg..

[5] Manyam B.V., Shah C., Woody N.M., Reddy C.A., Weller M.A., Juloori A., Naik M., Valente S., Grobmyer S., Durand P. (2019). Long-term outcomes after autologous or tissue expander/implant–based breast reconstruction and postmastectomy radiation for breast cancer. Pract. Radiat. Oncol..

[6] Schaverien M.V., Macmillan R.D., McCulley S.J. (2013). Is immediate autologous breast reconstruction with postoperative radiotherapy good practice?: A systematic review of the literature. J. Plast. Reconstr. Aesthetic Surg..

[7] Taghizadeh R., Moustaki M., Harris S., Roblin P., Farhadi J. (2015). Does post-mastectomy radiotherapy affect the outcome and prevalence of complications in immediate DIEP breast reconstruction? A prospective cohort study. J. Plast. Reconstr. Aesthetic Surg..

[8] Kaidar-Person O., Offersen B.V., Hol S., Arenas M., Aristei C., Bourgier C., Cardoso M.J., Chua B., Coles C.E., Damsgaard T.E. (2019). ESTRO ACROP consensus guideline for target volume delineation in the setting of postmastectomy radiation therapy after implant-based immediate reconstruction for early stage breast cancer. Radiother. Oncol..

[9] Song W.J., Kang S.G., Kim E.K., Song S.Y., Lee J.S., Lee J.H., Jin U.S. (2020). Current status of and trends in post-mastectomy breast reconstruction in Korea. Arch. Plast. Surg..

[10] Mandelbaum A., Nakhla M., Seo Y.J., Dobaria V., Attai D.J., Baker J.L., Thompson C.K., DiNome M.L., Benharash P., Lee M.K. (2021). National trends and predictors of mastectomy with immediate breast reconstruction. Am. J. Surg..

[11] Bustos V.P., Laikhter E., Manstein S.M., Comer C.D., Veeramani A., Shiah E., Xun H., Lin S.J., Lee B.T. (2022). A national analysis of outpatient mastectomy and breast reconstruction trends from 2013 through 2019. J. Plast. Reconstr. Aesthetic Surg..

[12] Dayaratna N., Nguyen C.L., Spillane A., Mak C., Warrier S.K., Dusseldorp J.R. (2023). Trends and variations in post-mastectomy breast reconstruction rates in Australia over 10 years. ANZ J. Surg..

[13] Sinnott C.J., Persing S.M., Pronovost M., Hodyl C., McConnell D., Ott Young A. (2018). Impact of postmastectomy radiation therapy in prepectoral versus subpectoral implant-based breast reconstruction. Ann. Surg. Oncol..

[14] Long C., Kraenzlin F., Aravind P., Kokosis G., Yesantharao P., Sacks J.M., Rosson G.D. (2022). Prepectoral breast reconstruction is safe in the setting of post-mastectomy radiation therapy. J. Plast. Reconstr. Aesthetic Surg..

[15] Weber W.P., Shaw J., Pusic A., Wyld L., Morrow M., King T., Mátrai Z., Heil J., Fitzal F., Potter S. (2022). Oncoplastic breast consortium recommendations for mastectomy and whole breast reconstruction in the setting of post-mastectomy radiation therapy. Breast.

[16] De Rose F., Fogliata A., Franceschini D., Cozzi S., Iftode C., Stravato A., Tomatis S., Masci G., Torrisi R., Testori A. (2019). Postmastectomy radiation therapy using VMAT technique for breast cancer patients with expander reconstruction. Med. Oncol..

[17] Doherty C., McClure J.A., Baxter N.N., Brackstone M. (2023). Complications From Postmastectomy Radiation Therapy in Patients Undergoing Immediate Breast Reconstruction: A Population-Based Study. Adv. Radiat. Oncol..

[18] Hall J., Fried D., Marks L.B., Gupta G.P., Jones E., Elmore S., Pearlstein K., Downs-Canner S., Gallagher K., Spanheimer P.M. (2022). Dosimetric and Clinical Factors Associated With Breast Reconstruction Complications in Patients Receiving Postmastectomy Radiation. Pract. Radiat. Oncol..

[19] De Sousa C.F.P.M., Neto E.S., Chen M.J., Silva M.L.G., Abrahão C.H., Ramos H., Fogaroli R.C., de Castro D.G., Favareto S.L., Pinto P.J.J. (2022). Postmastectomy Radiation Therapy Bolus Associated Complications in Patients Who Underwent 2-stage Breast Reconstruction. Adv. Radiat. Oncol..

[20] Chang J.S., Song S.Y., Oh J.H., Lew D.H., Roh T.S., Kim S.Y., Keum K.C., Lee D.W., Kim Y.B. (2019). Influence of radiation dose to reconstructed breast following mastectomy on complication in breast cancer patients undergoing two-stage prosthetic breast reconstruction. Front. Oncol..

[21] Naoum G.E., Ioakeim M.I., Shui A.M., Salama L., Colwell A., Ho A.Y., Taghian A.G. (2022). Radiation modality (Proton/Photon), timing, and complication rates in patients with breast cancer receiving 2-stages Expander/Implant reconstruction. Pract. Radiat. Oncol..

[22] Nava M.B., Pennati A.E., Lozza L., Spano A., Zambetti M., Catanuto G. (2011). Outcome of Different Timings of Radiotherapy in Implant-Based Breast Reconstructions. Plast. Reconstr. Surg..

[23] Cordeiro P.G., Albornoz C.R., McCormick B., Hudis C.A., Hu Q., Heerdt A., Matros E. (2015). What is the optimum timing of post-mastectomy radiotherapy in two-stage prosthetic reconstruction: Radiation to the tissue expander or permanent implant?. Plast. Reconstr. Surg..

[24] Ricci J.A., Epstein S., Momoh A.O., Lin S.J., Singhal D., Lee B.T. (2017). A meta-analysis of implant-based breast reconstruction and timing of adjuvant radiation therapy. J. Surg. Res..

[25] Santosa K.B., Chen X., Qi J., Ballard T.N., Kim H.M., Hamill J.B., Bensenhaver J.M., Pusic A.L., Wilkins E.G. (2016). Post-Mastectomy Radiation Therapy (PMRT) and Two-Staged Implant-Based Breast Reconstruction: Is There a Better Time to Radiate?. Plast. Reconstr. Surg..

[26] Momoh A.O., Griffith K.A., Hawley S.T., Morrow M., Ward K.C., Hamilton A.S., Shumway D., Katz S.J., Jagsi R. (2019). Patterns and correlates of knowledge, communication, and receipt of breast reconstruction in a modern population-based cohort of patients with breast cancer. Plast. Reconstr. Surg..

